# Speech motor planning and execution deficits in early childhood stuttering

**DOI:** 10.1186/s11689-015-9123-8

**Published:** 2015-08-20

**Authors:** Bridget Walsh, Kathleen Marie Mettel, Anne Smith

**Affiliations:** Department of Speech, Language, and Hearing Sciences, Purdue University, Lyles Porter Hall, 715 Clinic Dr., West Lafayette, 47907-2122 IN USA; University of Colorado Hospital, 12605 East 16th Ave., Aurora, CO USA

**Keywords:** Stuttering, Speech motor control, Preschool children, Speech kinematics, Speech production, Sex differences

## Abstract

**Background:**

Five to eight percent of preschool children develop stuttering, a speech disorder with clearly observable, hallmark symptoms: sound repetitions, prolongations, and blocks. While the speech motor processes underlying stuttering have been widely documented in adults, few studies to date have assessed the speech motor dynamics of stuttering near its onset. We assessed fundamental characteristics of speech movements in preschool children who stutter and their fluent peers to determine if atypical speech motor characteristics described for adults are early features of the disorder or arise later in the development of chronic stuttering.

**Methods:**

Orofacial movement data were recorded from 58 children who stutter and 43 children who do not stutter aged 4;0 to 5;11 (years; months) in a sentence production task. For single speech movements and multiple speech movement sequences, we computed displacement amplitude, velocity, and duration. For the phrase level movement sequence, we computed an index of articulation coordination consistency for repeated productions of the sentence.

**Results:**

Boys who stutter, but not girls, produced speech with reduced amplitudes and velocities of articulatory movement. All children produced speech with similar durations. Boys, particularly the boys who stuttered, had more variable patterns of articulatory coordination compared to girls.

**Conclusions:**

This study is the first to demonstrate sex-specific differences in speech motor control processes between preschool boys and girls who are stuttering. The sex-specific lag in speech motor development in many boys who stutter likely has significant implications for the dramatically different recovery rates between male and female preschoolers who stutter. Further, our findings document that atypical speech motor development is an early feature of stuttering.

## Background

Fluent speech production involves intricate and dynamic interactions among multiple neural systems governing cognitive, linguistic, emotional, motor, and perceptual aspects of speech production. We and others have adopted a multifactorial view that a combination of these domains is implicated in stuttering, a neurodevelopmental disorder which emerges in early childhood [1–4]. The hallmark characteristics of stuttering (i.e., sound repetitions, prolongations, and blocks) ultimately represent breakdowns in the precisely timed and coordinated articulatory movements required for fluent speech. Accordingly, there has been considerable experimental effort devoted toward understanding speech motor characteristics of adults who stutter (AWS). Collectively, these studies revealed subtle differences and instabilities in the relative timing, speed, and coordination of articulatory movements of AWS even during their production of perceptibly fluent speech [5–10].

An overarching question that has received little experimental attention, however, is whether these instabilities and differences in underlying speech motor dynamics observed in AWS are present near the onset of stuttering, in the preschool years, when most children who are stuttering ultimately will recover. There is sparse and often conflicting evidence that this is the case. For example, Chang et al. [11] found that young children who stutter (CWS) used slower articulatory movements (as inferred from acoustic measures) than children who do not stutter (CWNS). Conversely, Subramanian and Yairi [12] found no group differences in the same acoustic indicators of articulator speed in preschool CWS and CWNS. There also have been conflicting results concerning voicing and respiratory control during speech with several studies reporting differences between preschool CWS and CWNS [13–15] or alternatively, no group differences [16, 17]. A major limitation of these earlier studies is that most reported data from fewer than 10 CWS. Like AWS, CWS are heterogeneous and in the case of the preschool population, as noted above, include those children who will ultimately recover from stuttering as well as those who will persist.

As part of an ongoing project to investigate the physiological correlates of early stuttering, we recently completed two direct kinematic studies of articulatory motor control in preschool children. Both experiments employed a measure of the consistency of articulatory coordination, one for a complex sentence production task [18] and the other in a nonword production task [19]. CWS evidenced greater coordination variability for both nonword and sentence production than their CWNS peers revealing, for the first time, a potential lag in the development of speech motor control in young CWS close to onset. We extend this work by including measures reflecting multiple aspects of speech motor control processes to more precisely characterize speech motor dynamics in preschool CWS. As nearly all of the participants in this relatively large-scale project completed the simpler sentence production tasks for the current report (compared to the two earlier studies from our laboratory), this experiment also includes data from a larger number of preschool CWS and their peers. Thus, we not only examine differences between CWS and CWNS but can determine if there are subgroups of preschool CWS who differ on these measures.

### Neural bases of stuttering

Many accounts of the neural bases of stuttering attribute deficient speech motor planning and execution and auditory and sensorimotor integration to breakdowns in speech fluency [20–24]. Support for these assertions comes, in part, from nearly two decades of neuroimaging research implicating subtle structural and functional differences in the neural networks supporting speech production in adults who stutter [25–36]. In recent years, there have been a few structural neuroimaging studies in CWS that have also revealed diffuse and heterogeneous gray and white matter differences in CWS compared to CWNS in neural regions integral to fluent speech production. The findings in CWS, however, do not parallel the neuroanatomical profiles of AWS [37–40]. Thus, while atypical structure and function of neural systems supporting speech planning and execution are implicated in stuttering in both children and adults, future neuroimaging efforts in CWS would clearly be aided by specification of what speech motor deficits do or do not characterize early stuttering in preschoolers.

### Characteristics of speech motor control in typically fluent children

Earlier, relatively large-scale, cross-sectional studies of groups of typically fluent children spanning age 4 years through young adults reveal that the typical pattern of speech motor development is protracted, with adult-like speech motor dynamics not appearing until the late teen years [41–44]. Young children use an immature strategy of speech production characterized by large articulatory displacements relative to their smaller orofacial structures (quantified through anthropometric measurement) albeit at lower velocities and longer durations compared to adults [41, 43, 45]. We hypothesized that young children employed this strategy to enhance auditory and somatosensory feedbacks both of which are critical to the development and maintenance of stable speech motor programs (e.g., [46, 47]).

In addition to basic measures of displacement and velocity, a measure that has proven effective in documenting the course of speech motor development is the lip aperture (LA) index, which captures trial-to-trial variability in the interactions among both central and peripheral processes involved in coordinated motions of the upper lip, lower lip, and jaw for repeated productions [42]. Articulatory movements of the lips and jaw must be precisely coordinated to accomplish the dynamic control of lip aperture size and shape, a vocal tract parameter with significant effects on the speech acoustic signal [48]. Although the articulators can achieve oral opening and closing through many different movement configurations, when adults are asked to repeat a sentence, they show highly consistent articulatory patterns, reflecting stable underlying muscle synergies that effectively reduce the degrees of movement freedom associated with this task [42, 49]. The articulatory patterns of young children are highly variable from trial-to-trial [42, 43], and the extended course of speech motor development most likely reflects a motor system adapting to dramatic developmental changes at multiple levels, from the growth of orofacial structures to the maturation of the neural networks supporting language and speech functions.

It has been proposed that AWS rely to a greater extent on immature, slower, and less efficient feedback-based speech motor control mechanisms due to the faulty formation of stable internal representations or speech motor commands [21, 24, 50]. In the present study, fundamental indices of speech motor control (i.e., movement duration, amplitude, and velocity) as well as a dynamic measure that captures the overall consistency of articulatory coordination are used to ascertain whether CWS, close to stuttering onset, operate with a less mature speech motor control system compared to their nonstuttering peers. Specifically, we predict that the articulatory profiles of CWS will be characterized by decreased interarticulator coordination, longer durations (indicative of slower speaking rates), and larger amplitude/lower velocity articulatory movements compared to CWNS. This constellation of traits distinguishes the speech motor performance of typically fluent preschool-aged children from that of older children and adults, and we hypothesize that CWS will show less mature speech motor performance to an even greater extent than their nonstuttering peers.

## Methods

### Participants

Data collection was carried out at two sites: the Department of Speech, Language, and Hearing Sciences, Purdue University and the Department of Communication Sciences and Disorders, University of Iowa. The research protocols were conducted with the approval of the Institutional Review Boards of both Universities. Written informed consent was obtained from all parents/legal guardians during the initial testing session. Fifty-eight CWS (44 boys, 14 girls) and 43 age-matched CWNS (29 boys, 14 girls) participated in the study. All participants were between 4;0 (years; months) and 5;11 (CWS, *M* = 4;8, SD = 7 months); (CWNS, *M* = 4;8, SD = 6 months).

All children were native speakers of North American English with normal hearing and no history of neurological disorders. As part of a larger experimental protocol, a comprehensive battery of speech and language assessments was administered to each child. These included measures of speech production ([51]; consonant inventory of the Bankson-Bernthal Test of Phonology BBTOP-CI), expressive language ([52]; Structured Photographic Expression of Language Test Third Edition SPELT-3), and receptive language ([53]; Test for Auditory Comprehension of Language Third Edition TACL-3). All CWNS had to pass (i.e., obtain a standard score of 85 or better) on these assessments in order to participate in the study. However, we included all CWS in order to reflect the heterogeneity of this population [54, 55]. Table 1 provides descriptive data regarding performance on these measures. In order to be eligible for the study, *all* children needed to score within normal limits on assessments of nonverbal intelligence (CWS, *M* = 112, SD = 10; CWNS, *M* = 112, SD = 10) ([56]; Columbia Mental Maturity Scale) and social development (CWS, *M* = 17, SD = 2; CWNS, *M* = 16, SD = 2) ([57]; Childhood Autism Rating Scale). Additionally, the two groups had comparable socioeconomic status (both *M* = 6, SD = 1) determined by their mothers’ level of education in their first year in the study ([58]; 1 = less than seventh grade education through 7 = graduate degree).

**Table 1:** Performance on standardized speech and language tests

	BBTOP-CI			SPELT-3			TACL-3		
	Range	Mean	SD	Range	Mean	SD	Range	Mean	SD
CWS M	65–114	90	14	63–121	98	14	76–143	111	15
CWS F	72–115	96	12	82–122	101	11	100–126	112	10
CWNS M	87–118	101	10	88–127	110	10	91–143	119	16
CWNS F	95–117	103	8	86–130	111	12	96–141	122	11

### Stuttering diagnosis

Participants were diagnosed as CWS using the three criteria established by Ambrose and Yairi [59] and Yairi and Ambrose [60] which include the diagnosis by a speech-language pathologist, clinical and parental ratings of severity, and analyses of disfluencies in 750–1000 word samples of spontaneous speech. The average age of stuttering onset was 35 months (SD = 9 months) and duration of stuttering (i.e., time since onset) was 22 months (SD = 9 months) according to parent report. The CWS ranged in severity from very mild to severe. Approximately 54 % of the cohort had a mild, 40 % a moderate, and 6 % a severe stuttering problem. Finally, CWS were eligible to participate in the study regardless of whether they had received or were currently receiving speech therapy. We documented that approximately 39 % of the boys who stutter (17/44) and 36 % of the girls who stutter (5/14) had received speech therapy.

### Task and procedures

Participants spoke aloud two simple-structured sentences, (“Buy Bobby a puppy” and “Mommy bakes potpies”) which contain consonants to target superior-inferior upper lip and lower lip (plus jaw) movement (only the movement of these articulators was tracked). These sentences contain age-appropriate phonemes typically acquired between 36–42 months [61] and grammatical morphemes—Stage III of Brown’s stages of language development typically acquired between 31–34 months [62]. Children were excluded if they made errors on any phoneme in the two sentences. Participants repeated the sentence “Buy Bobby a puppy” in response to a recorded model spoken by an adult female speaker of North American English. This sentence was randomized with longer and more complex sentences as a part of a larger experimental protocol. Next, in a fluency enhancing condition, we recorded successive repetitions of the sentence “Mommy bakes potpies.” In this case, the experimenter initially modeled the sentence for the child and then cued him/her to produce it independently. Each time the child repeated the sentence, s/he earned a toy to add to a chain until at least 10 productions were obtained. The participants practiced saying each sentence at least two times (but no more than three times) before data collection began. They were instructed to use their “regular talking voice” when producing the sentences. Only accurate and fluent tokens of each sentence were used in the analyses. A sentence was judged to be acceptable when it did not contain substitutions, omissions, additions, any disfluency, aberrant prosody, or inappropriate pauses. This was done during the session by one experimenter and confirmed later by a second experimenter during offline data analysis.

### Apparatus

Kinematic data were collected with a Northern Digital Optotrak 3020 movement tracking system. The cameras record the three-dimensional movements of small infrared light emitting diodes (IREDs) adhered to the lips with electrode collars. One IRED was affixed to the vermilion border of the upper lip at midline and one to the center of the lower lip. To eliminate artifact, from head movement for example, five additional IREDs were used to compute a head coordinate system for each participant. Superior-inferior upper lip and lower lip movements were then transduced relative to this head coordinate system [43]. Motion of each IRED was digitized at 250 Hz. The participant’s acoustic signal was collected with a condenser microphone and digitized at a 16-kHz sampling rate by an A/D unit within the Optotrak system so that it was synchronized with the movement signals.

### Kinematic data analysis

**Fig. 1:**
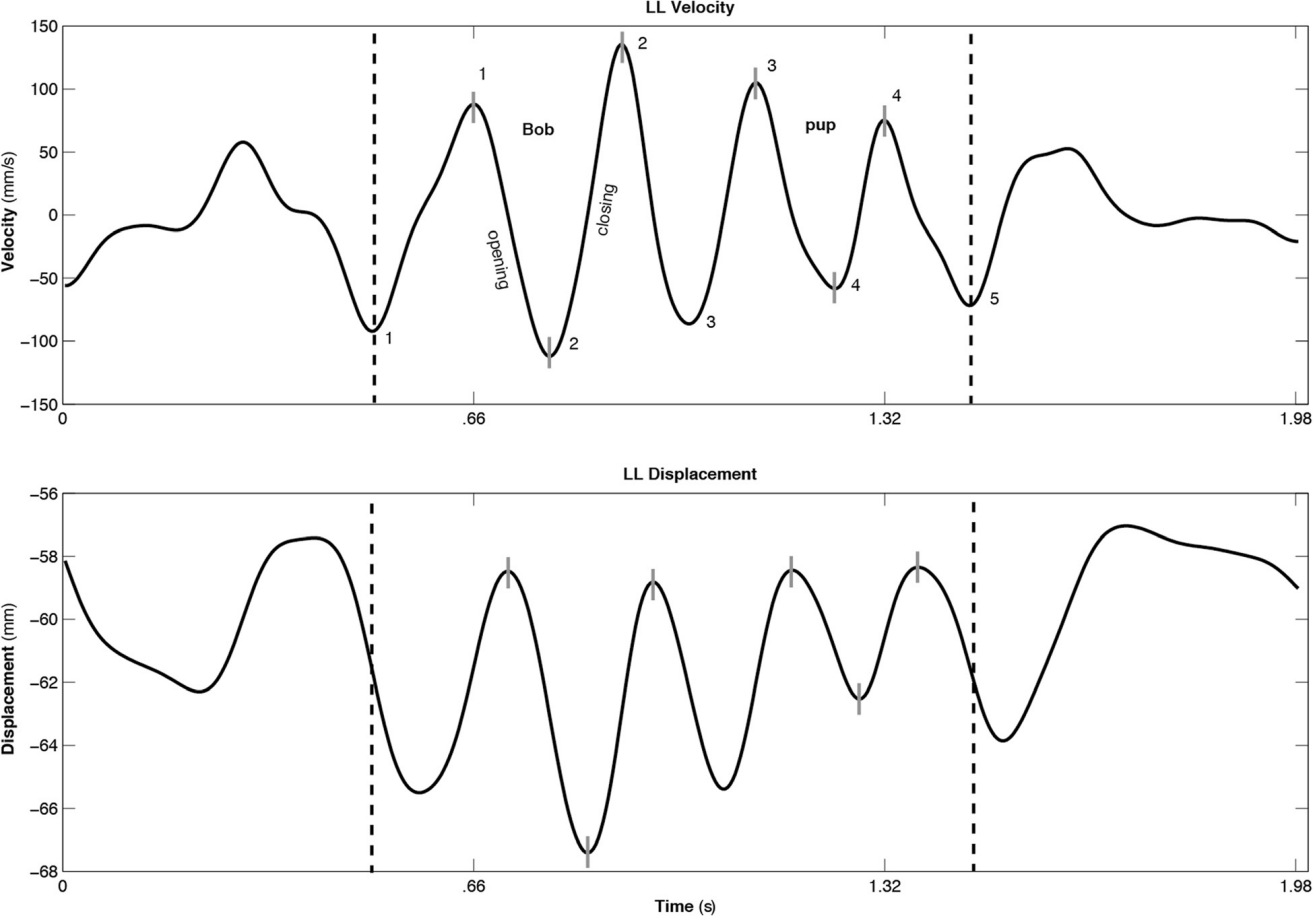
Lower lip velocity and displacement traces from one production of “Buy bobby a puppy” from a 5-year-old CWNS. *Dashed vertical lines* pass through the first and fifth negative opening peak velocities to show how each lower lip and upper lip (not shown) displacement trajectories were segmented for phrase level analyses. *Short solid lines* on the traces indicate the selection of the opening and closing movements to produce the syllables “Bob” and “pup” for the single movement analyses

We included between 7 and 10 accurate and fluent iterations of each sentence from each participant in the kinematic analyses (practice trials and first productions were discarded and up to 10 out of a possible 12 total acceptable productions were utilized) [18]. Consistent with established methods [42, 43, 63], a custom MATLAB (The Mathworks) script displayed the displacement and velocity signals from each sentence repetition on a computer monitor. The lower lip velocity signal was used to segment the upper and lower lip trajectories from the beginning and end points of each repetition; in this case, the first negative peak velocity associated with the first opening movement of the sentences (release of the /b/ in the word “buy” or /m/ in the word “mommy”) to the fifth negative opening peak velocity (release of the /p/ to /i/ in “puppy” or /p/ to the vowel in “pies” (Fig. 1). The synchronized audio signal was used to verify that the target sentences were produced accurately and that they were not inadvertently cut off during segmentation.

### Dependent variables

#### Single movement, basic kinematic parameters

Measures of peak opening and closing displacement amplitude, velocity, and duration of the lower lip (plus jaw) articulatory movements associated with a relatively larger oral opening target (the word “Bob” in the sentence “Buy Bobby a puppy”) and a relatively smaller oral opening target (“pup” in the same sentence) were computed to characterize articulatory movement for these internal components of the sentence. As shown in Fig. 1, a custom-written MATLAB script automatically extracted the opening-closing movement sequences for “Bob” and “pup” from the original segmented “Buy Bobby a puppy” displacement and velocity waveforms.

#### Phrase level displacement and velocity measurements

The following measures were used to assess articulatory movement for sentence production. The displacement dynamic range and the velocity dynamic range comprised 80 % of points across the displacement and velocity trajectories. These measures capture the primary operating range of the lower lip/jaw for the whole utterance [41, 64]. One displacement and one velocity dynamic range was computed for each repetition of the sentence “Buy Bobby a puppy” then an average was taken for each participant.

#### Phrase level duration measure

**Fig. 2:**
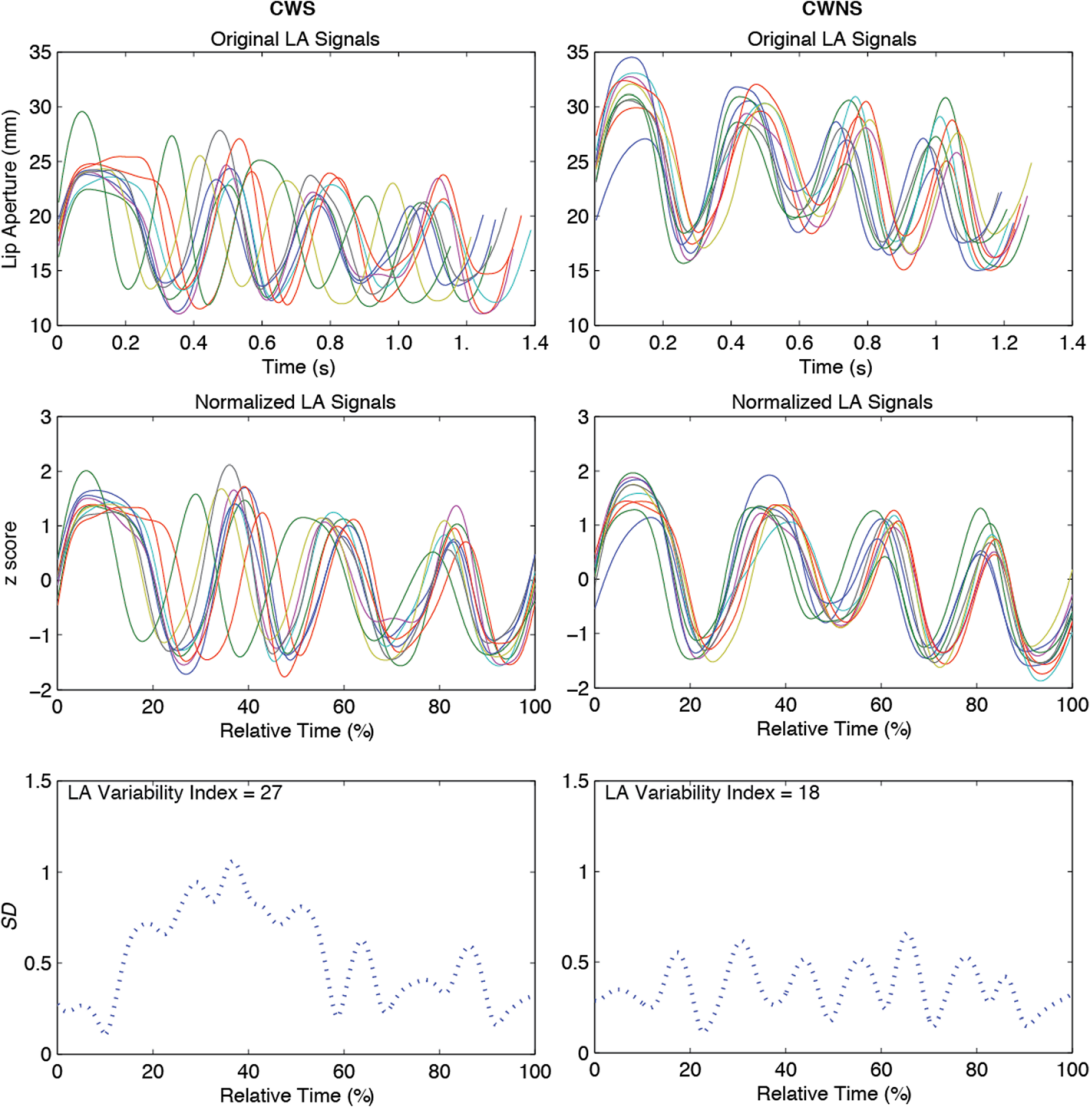
Lip aperture (LA) variability index calculation. The *top panels* show 10 LA displacement trajectories form one male CWS (*left*) and one female CWS (*right*) participant repeating “Buy Bobby a puppy.” In the *middle panels*, the 10 traces have now been time- and amplitude-normalized. The *bottom panels* show the standard deviations of the 10 normalized traces computed successively at 2 % intervals in relative time. The sum of these standard deviations, the LA variability index, is shown in the *bottom panels*

The sentence duration of the lower lip movement sequence for each sentence was computed for each participant as the time in seconds of each original, nonnormalized lower lip sentence repetition after segmentation (Fig. 2, top panel). The set of duration values for each sentence was then averaged for each participant.

#### Phrase level coordination measure

The lip aperture (LA) index was calculated by a sample-by-sample subtraction of the segmented lower lip displacement signal (see above) from the segmented upper lip displacement signal for the sentences “Buy Bobby a puppy” and “Mommy bakes potpies.” Thus, the LA signal represents the coordination of the upper lip, lower lip, and jaw to control oral opening and closing across these sentences (e.g., [42]). Figure 2 shows this calculation for one CWS and CWNS. The lip aperture trajectories from each participant (7–10 trials for each type of sentence were then time-normalized with a cubic spline procedure to project each displacement trajectory onto a consistent axis length of 1000 points (middle panel in Fig. 2). Also shown in this panel, the trajectories were amplitude-normalized by subtracting the mean of the lip aperture displacement signal and dividing by its standard deviation. Finally, the standard deviation across all trajectories was computed at fixed 2 % intervals in relative time and then summed producing the LA index (Fig. 2, bottom panel). Higher values of the LA index reflect greater variability.

## Results

As detailed in the “Methods” section, only data from participants who produced at least seven fluent and accurate productions of a sentence were included in the analyses. The number of children who produced the requisite number of trials for “Buy Bobby a puppy” was 57 (43 boys and 14 girls) for the CWS and 40 (27 boys and 13 girls) for the CWNS. For the sentence “Mommy bakes potpies”, we included data from 51 CWS (39 boys and 14 girls) and 40 (26 boys and 14 girls) CWNS. Three subjects’ data were omitted due to obvious errors in the calculation of the dynamic range measures (values exceeded the adult normative range obtained from an earlier study by 3 SD; 41). A Levene’s test was calculated for each ANOVA to ensure that the groups had approximately equal variance (all *p* between 0.39–0.94). The means and standard deviations of each dependent variable are provided in Table 2.

**Table 2:** Means and standard deviations for dependent variables by group

Group means (SD)								
Single movement measures	CWS-male		CWS-female		CWNS-male		CWNS-female	
	Open	Close	Open	Close	Open	Close	Open	Close
LL displacement (mm) Bob	8.4 (2.9)	7.4 (2.4)	9.1 (3.2)	8.2 (3.0)	10.0 (2.8)	8.8 (2.7)	8.8 (3.3)	8.1 (3.0)
LL displacement (mm) pup	6.2 (2.3)	6.3 (2.0)	6.5 (2.3)	6.4 (2.2)	7.1 (2.0)	7.2 (2.1)	6.1 (2.2)	6.4 (2.6)
LL velocity (mm/s) Bob	95.9 (36.4)	104.4 (36.6)	109.2 (46.3)	114.3 (41.1)	118.4 (36.8)	121.7 (37.1)	100.0 (39.3)	115.0 (43.9)
LL velocity (mm/s) pup	76.1 (32.8)	88.3 (28.3)	81.2 (36.4)	93.6 (33.0)	91.8 (30.2)	105.4 (31.4)	77.8 (30.5)	93.1 (38.3)
Syllable duration (s) Bob	0.16 (0.02)	0.12 (0.02)	0.15 (0.02)	0.12 (0.02)	0.14 (0.02)	0.13 (0.02)	0.15 (0.01)	0.12 (0.01)
Syllable duration (s) pup	0.14 (0.02)	0.15 (0.15)	0.14 (0.02)	0.12 (0.02)	0.13 (0.02)	0.12 (0.02)	0.13 (0.02)	0.12 (0.02)
Phrase measures	CWS-male		CWS-female		CWNS-male		CWNS-female	
Displacement dynamic range (mm)	8.3 (2.4)		9.4 (3.2)		10.0 (2.5)		8.6 (2.5)	
Velocity dynamic range (mm/s)	146.5 (42.8)		172.5 (56.4)		188.2 (47.8)		160.7 (51.3)	
LA index BBAP	25.3 (4.6)		20.6 (5.6)		22.3 (4.1)		20.9 (4.2)	
Duration BBAP (s)	1.2 (0.2)		1.2 (0.1)		1.1 (0.1)		1.1 (0.07)	
LA index MBPP	25.2 (5.0)		20.3 (5.0)		22.4 (5.4)		21.6 (4.6)	
Duration MBPP (s)	1.3 (0.2)		1.3 (0.2)		1.4 (0.3)		1.3 (0.2)	

*LL* lower lip, *BBAP* Buy Bobby a puppy, *MBPP* Mommy bakes potpies

### Single movement measurements: displacement, velocity, and duration

Separate ANOVAs with repeated measures on movement direction (2: opening and closing) and syllable (2: Bob and pup) were computed to assess group and sex effects on displacement amplitude, velocity, and duration. CWS and CWNS groups had similar displacement amplitudes *F*(1,93) < 1, and the sex effect was not significant *F*(1,93) < 1. As expected based on our earlier work [43], the effects of word *F*(1,91) = 55.58, *p* < 0.001 and direction *F*(1,93) = 19.72, *p* < 0.001 were significant. “Bob” was associated with larger amplitude movements compared to “pup,” and opening displacements were consistently larger compared to closing.

There were no stuttering or sex group differences for opening and closing peak velocity for production of these syllables both *F* (1,93) < 1. The effect of word *F*(1,93) = 70.05, *p* < 0.001 and direction *F*(1,93) = 8.95, *p* < 0.001 were significant. Bob was associated with higher velocities compared to pup, while closing articulatory movement velocities are typically higher than opening velocities [6, 64].

Finally, the duration of opening and closing articulatory movements associated with syllable production were similar for CWS and CWNS *F* (1,93) < 1. There was no sex effect for this measure *F*(1,93) < 1. The effect of syllable *F*(1,93) = 19.10, *p* < 0.001 and direction *F*(1,93) = 131.61, *p* < 0.001 were significant. There were significantly longer opening and closing durations for the syllable “Bob” compared to “pup,” while opening articulatory movements were typically longer than closing movements [64].

### Phrase level measurements: displacement, velocity, duration, and coordinative variability

#### Displacement and velocity dynamic range

**Fig. 3:**
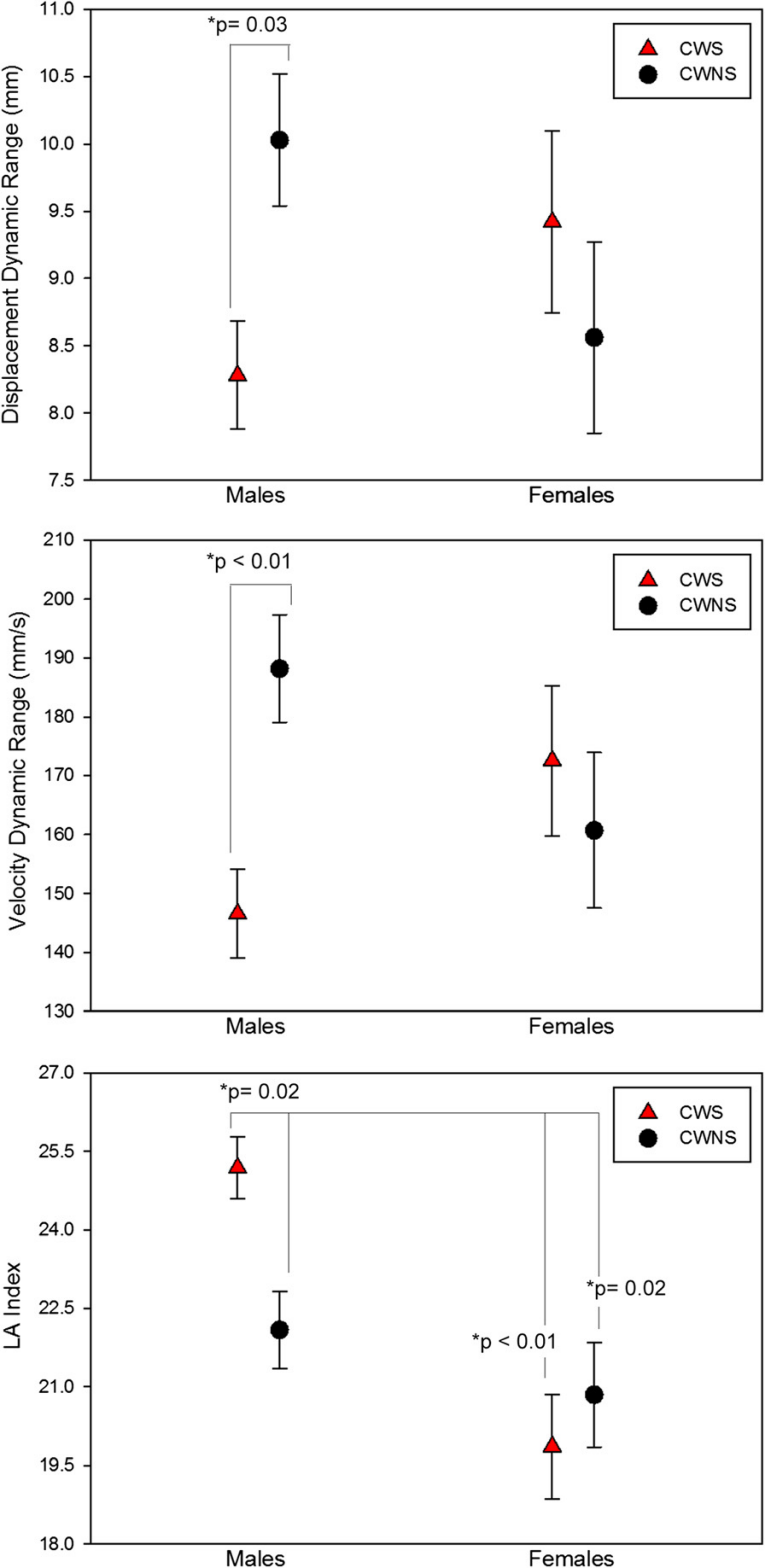
The average displacement (*top panel*) and velocity (*middle panel*) dynamic ranges and LA variability index (*bottom panel*) with standard error bars for each group. Male CWS operated with significantly reduced articulatory displacement and velocity compared to male CWNS. Male CWS also had higher coordinative variability than all the other groups of children

**Fig. 4:**
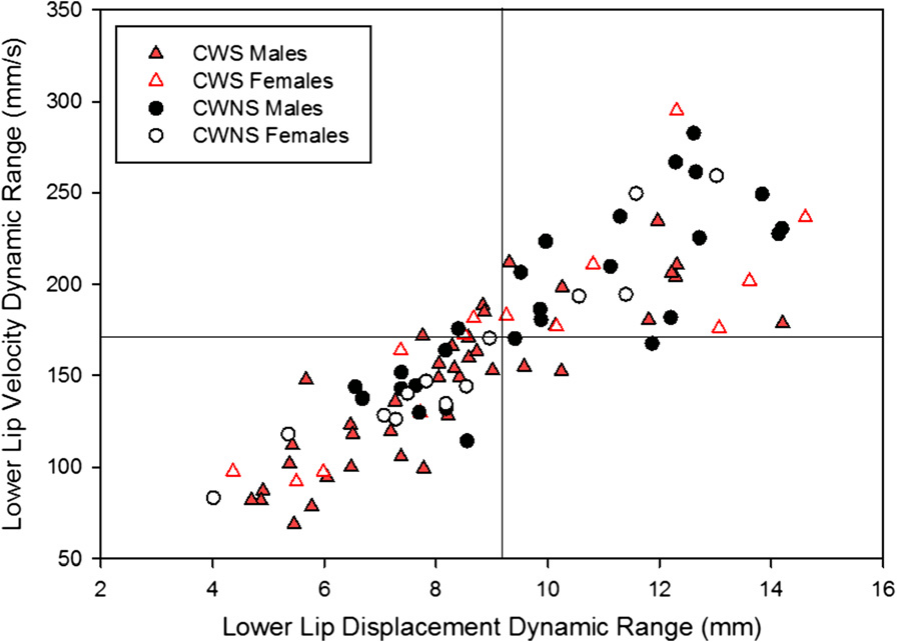
Dynamic ranges for “Buy Bobby a puppy.” Data points from each participant reveal their average lower lip displacement dynamic range plotted against their average velocity dynamic range. The *vertical* and *horizontal lines* in this graph show the median value for the dynamic ranges for the CWNS. The *lower left* quadrant of the graph contains data from participants with the smallest displacement and lowest velocity dynamic ranges. Conversely, the *upper right* quadrant contains data from those participants with the largest displacement and highest velocity dynamic ranges

Figure 3 includes graphs showing mean group data for both dynamic range measures. A two-way ANOVA did not reveal significant stuttering or sex group effects for the displacement dynamic range measure, both *F*(1,90) < 1. However, the sex by stuttering interaction *F*(1,90) = 5.02, *p* = 0.03 was significant. Similarly, the stuttering and sex group effects were not significant for the velocity dynamic range; both *F*(1,90) < 1. But the sex by stuttering interaction *F*(1,90) = 6.0, *p* = 0.02 was significant for this measure. Post hoc comparisons (HSD for unequal *N*) confirmed that male CWS had significantly reduced displacement dynamics ranges *p* = 0.03 and velocity dynamic ranges *p* < 0.01 compared to male CWNS. As shown in the top and middle graphs of Fig. 3, with the exception of the male CWS/CWNS comparison, the other group comparisons were not significantly different (all *p* between 0.3–0.9). The individual data presented in Fig. 4 shows each participant’s displacement dynamic range plotted against his/her velocity dynamic range according to their respective group (male and female CWS and CWNS). The graph is divided into quadrants by plotting the median for the control groups for each dynamic range. The lower left quadrant of the graph reveals data from participants with the smallest movement amplitude and lowest velocity operating ranges, while the upper right quadrant contains data from participants with the largest displacement amplitudes and highest velocities. Although this graph reveals overlap among the groups, approximately 67 % of the male CWS are clustered in the lower left section.

#### Duration

An ANOVA with repeated measures on sentence duration (2) did not reveal significant group effects of stuttering or sex nor was the interaction significant for this measure (all *F*(1,83) < 1). There was an effect of sentence *F*(1,83) = 41.3, *p* < 0.001. On average, the sentence “Mommy bakes potpies” was longer than “Buy Bobby a puppy” by approximately 0.20 s.

#### Lip aperture index

**Fig. 5:**
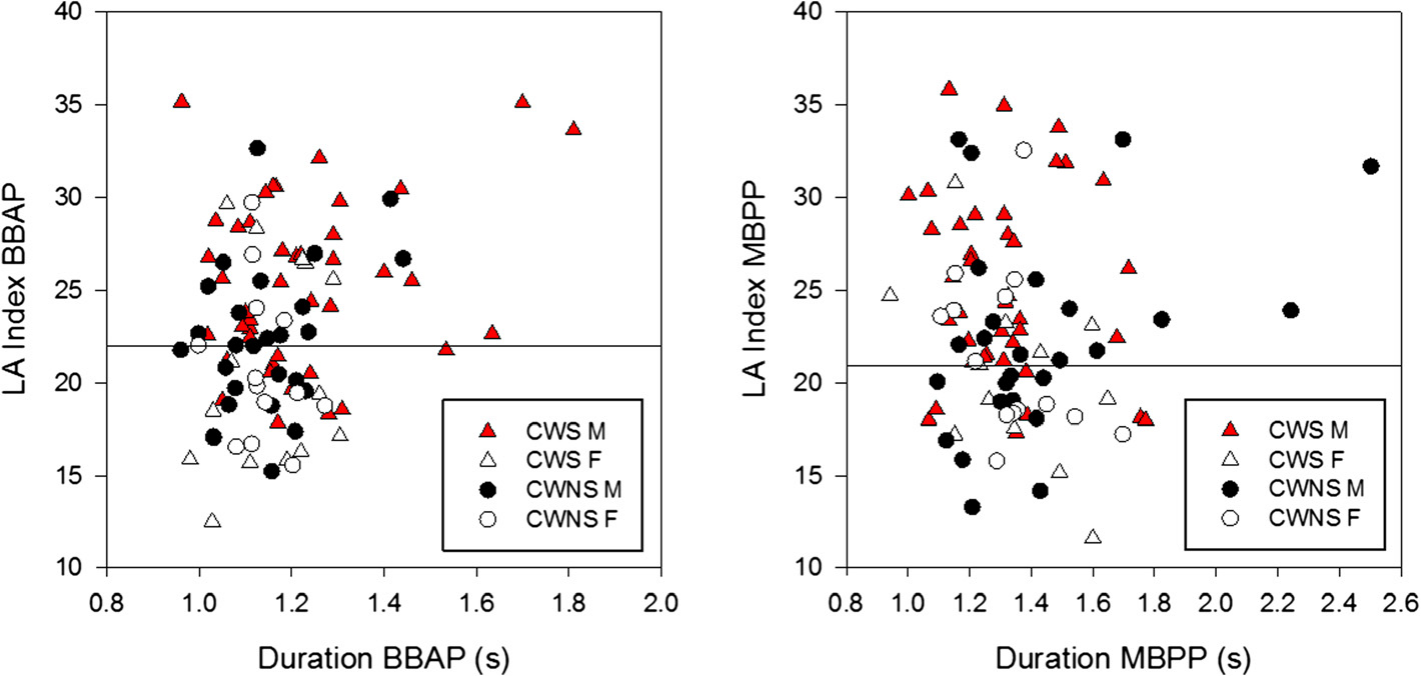
An average LA variability index for each participant is plotted against his/her average duration for “Buy Bobby a puppy” (BBAP-*left*) and “Mommy bakes potpies” (MBPP-*right*). The *horizontal lines* in each graph show the median LA index for the CWNS. Higher scores on the *y*-axis are associated with greater articulatory movement variability

An ANOVA with repeated measures on sentence (2) did not reveal a significant stuttering group effect *F*(1,83) < 1, but there was a significant sex effect *F*(1,83) = 14.5, *p* < 0.001. Males (*M* = 24.0) had higher LA indexes (denoting greater variability) than females (*M* = 20.37). But there was also a significant sex effect by stuttering interaction for this measure *F*(1,83) = 5.6, *p* = 0.02. As the bottom graph of Fig. 3 shows, post hoc comparisons (HSD for unequal *N*) revealed that male CWS had significantly higher LA indices compared to all the other groups of children. The other groups, however, were not significantly different from each other (all *p* between 0.4–0.9). In Fig. 5, each child’s average LA index for each sentence is plotted against his/her average duration for that sentence. The horizontal lines on each graph show the median LA index from the control group of children for each sentence. Thus, participants with the lowest LA variability indexes (denoting greater stability) fall below this line, while participants with the highest LA scores (higher variability) fall above. As the statistics revealed, 72 % of the boys who stuttered had LA indexes above the median line for “Buy Bobby a puppy” (82 % for “Mommy bakes potpies”) indicating high degrees of coordinative variability.

### Relationships among measures

We examined potential relationships among performances on the speech motor dependent variables (i.e., displacement and velocity dynamic ranges and average LA index) and other characteristics in the group of CWS including the following: stuttering severity and scores on speech and language assessments. As reported in Table 3, we calculated the correlations among all pairs of these measures. No pair of variables significantly correlated at the *p* = 0.05 level. For the history of therapy, independent sample *t* tests were used to test whether there were differences in performance on these three dependent variables between CWS who did and did not receive stuttering therapy. None of these tests were significant, all *t*(55) = 0.09–1.0, all *p* > 0.05.

**Table 3:** Correlations between standardized measures and variables of interest for CWS (*n* = 53; all correlations nonsignificant at the *p* = 0.05 level)

	Displacement dynamic range	Velocity dynamic range	Average LA index
Stuttering severity index	−0.16	−0.06	0.22
SPELT-3	0.13	−0.05	−0.22
TACL-3	0.13	0.05	−0.10
BBTOP-CI	0.06	−0.03	−0.05

## Discussion

We examined intrinsic characteristics of speech motor control processes during fluent speech production in CWS close to stuttering onset to evaluate the hypothesis that children recently diagnosed as stuttering would lag their peers on fundamental indices of speech motor development. Our results from a relatively large number of CWS showed that many of the boys who stutter, but not girls, produced fluent speech with reduced amplitudes and velocities of articulatory movement, as evidenced by smaller overall amplitude and velocity dynamic ranges, across sentence production. There were no differences among any of the groups on the overall duration of either single articulatory movements or phrase level productions, suggesting that the present findings are not driven by speech rate differences. Finally, we found that boys, particularly boys who stutter, used more variable combinations of articulator coupling to achieve dynamic lip aperture targets compared to girls, suggesting that boys who are stuttering have less mature speech coordinative patterns. This study is the first to demonstrate sex-related differences in speech motor control processes for preschool boys compared to girls who are stuttering, and therefore, the first to provide important evidence concerning the dramatically different ultimate recovery rates between preschool boys and girls who stutter.

### Displacement, velocity, and duration of articulatory movements

Based on findings from normative developmental studies, we anticipated that CWS would produce articulatory movements at reduced velocities; however, we did not predict that well over half of the boys who stutter would also produce smaller articulatory displacements compared to the other participants (Fig. 4). In a recent large-scale EMG study, we found that the amplitude and bilateral synchrony of perioral muscle activation patterns were similar for male and female preschool CWS and CWNS for sentence and spontaneous speech production [65]. These findings argue against a biomechanical limitation or muscular insufficiency preventing these children from speaking with velocities and displacements comparable to the other children from our sample. One possibility suggested in earlier studies of adults [10, 66] is that reduced velocities reflect the effect of treatment for stuttering, as slowing speech rate is a common therapeutic strategy. There were, however, no differences between groups in phrase level durations, indicating equivalent overall speech rates. In addition, the majority of our participants had not received speech therapy, and we did not find a significant effect of speech therapy on any of the speech production measures, so it is unlikely that the boys who stutter were employing a fluency enhancing technique during the speech tasks. Rather, our results implicate immature patterns of neural drive to the muscles in early stuttering even during fluent speech production. It is plausible that differences in developing speech neural networks in CWS, as revealed by recent neuroimaging investigations, affect the efficient formulation and transmission of these speech motor plans to the periphery [37–39].

Our findings concerning utterance durations are consistent with results from other speech rate studies of preschool CWS (e.g., [67, 68]). The majority of preschoolers across all groups produced speech at slower rates (indicated by longer durations) compared to older children and adults from our earlier investigations. The combined mean sentence durations (measured as the duration of the total kinematic record for the sequence) from children in the present study (*M* = 1.24 s) closely match durations on these same sentences from preschoolers published in earlier reports (*M* = 1.35 s) and are approximately 27 % longer, on average, than adult productions (*M* = 0.91 s) [42, 43]. We have suggested that slower speaking rates allow additional time for language formulation and speech motor planning and could also indicate greater reliance on slower, less efficient feedback control processes. It is interesting and relevant to note that the boys who stuttered had comparable sentence durations to the other participants despite having, on average, reduced operating ranges. Precise timing of rapid sequential movements of multiple articulators is critical for intelligible speech production and varying temporal cues in the speech acoustic signal significantly impairs speech perception [69]. Perhaps moving at lower velocities, albeit over smaller distances, allowed the boys who stuttered to preserve the relative timing of speech movements within and across sentences and thus optimize intelligibility.

We should also note, while the dynamic range measures revealed sex/group differences, parallel results were not observed for the single movement measures. The dynamic range measures characterize the primary “operating range” of the articulators, in contrast to the parameters associated with single, component movements. Thus, the present results suggest that the dynamic range measures are more reflective of the overall operational characteristics of the speech motor system that are not necessarily observable at the single movement level.

### Articulatory coordination

The lips and jaw can produce many different movement configurations to dynamically control oral opening and closing to achieve phonetic targets (e.g., [70]). With maturation during the childhood years, we see highly variable patterns of production decrease as speakers transition to more stable, mature articulatory patterns or movement synergies across development [42, 43]. In our earlier investigations [18, 19], we found greater coordinative variability during the production of nonwords and syntactically complex sentences in CWS compared to CWNS but did not have enough participants in each group to examine sex differences. In the present study, all of the preschoolers produced the sentences in a perceptually accurate and fluent manner; however, we found that boys, in particular boys who stutter, used more variable combinations of articulator coupling even for simple sentence production. This finding suggests a lag in the development of neural control and coordination of articulatory movements that is most pronounced in boys who stutter. Smith and Zelaznik [42] provided initial evidence of different developmental trajectories of speech motor control processes for preschool boys and girls, with 4- and 5-year-old girls demonstrating significantly lower LA variability compared to boys. By age 7, the effect of sex was not significant nor was it significant in the older age group comparisons, suggesting that boys “catch up” after this initial lag in speech motor coordination.

It is well established that more school-aged and adult males stutter than females [71, 72]. At stuttering onset (average age of onset is approximately 34 months), the ratio of males to females is estimated to be approximately 1.5:1 [73]. The male to female ratio increases to 3:1 for school-aged children and is estimated to be 4:1 to 6:1 for adults [71]. Clearly, many more girls recover from stuttering. Importantly, no differences in the characteristics of stuttering near onset (for example in severity or abruptness of onset) have been documented for girls compared to boys in relation to ultimate recovery or persistence of stuttering for either sex [59, 60]. Finally, related to recovery, the group differences we report here for the stuttering boys are compelling in light of the fact that at this age approximately 50 % of them will recover with or without treatment.

Preschool-aged boys are also more susceptible to other developmental disorders such as autism [74], speech delay [75], specific language impairment (e.g., [76]), and phonological impairment [75]. It is often suggested that sex differences in brain morphology such as in white matter tract characteristics [77], gray matter volume [78, 79], and the slope of neurological growth curves [80, 81] underlie the greater prevalence of these neurodevelopmental disorders among boys. With regard to stuttering, diffuse differences within speech neural networks could induce instability in the formulation and implementation of speech motor programs resulting, by implication, in more variable articulator coupling. It is important to note that the majority of girls in our stuttering group, unlike the boys, exhibited articulatory characteristics that were on par with their peers. It seems reasonable to suggest that the earlier maturation of central speech motor control networks in girls who stutter compared to boys (reflected by their more consistent articulatory patterning and age-appropriate displacement and velocity operating ranges) is a significant factor in the greater probability for girls to recover from stuttering. Having a more stable speech motor system may lend the girls an advantage as complex, emergent central nervous system networks manage the many different motor, cognitive, linguistic, and emotional demands that collectively interact during speaking. In future studies, it will be important to determine if both preschool boys and girls who stutter with relatively high coordination variability indices are more likely to persist in stuttering.

## Conclusions

Studies of young CWS are critical to inform models of stuttering which are almost exclusively based upon data from AWS. Novel findings from this study contribute to an emerging picture of stuttering close to its onset. Our fundamental and dynamic measures of articulatory characteristics collectively suggest that speech motor performance, particularly in boys who are stuttering, deviates from their peers who do and do not stutter even for the production of simple sentences. Critical questions remain, however, whether or not the speech motor differences we observed are early markers of persistent stuttering and regarding the time course of speech motor development in those children who demonstrated a delay in the development of speech motor processes. We have followed many of these children for up to 5 years, and as a result, we have data when they are older and stuttering recovery statuses are known. Follow-up, retrospective analyses are critical to determine whether higher variability in articulatory coupling and reduced velocity and displacement dynamic ranges at 4–5 years are precursors of persistent developmental stuttering.

## Abbreviations

AWS: Adults who stutter
CWNS: Children who do not stutter
CWS: Children who stutter
IREDs: Infrared light emitting diodes
LA index: Lip aperture index
